# Effects of Different Oxidation Degrees of Graphene Oxide on P-Type and N-Type Si Heterojunction Photodetectors

**DOI:** 10.3390/nano8070491

**Published:** 2018-07-04

**Authors:** Ching-Kuei Shih, Yu-Tang Ciou, Chun-Wei Chiu, Yu-Ru Li, Jia-Syun Jheng, Yen-Chun Chen, Chu-Hsuan Lin

**Affiliations:** Department of Opto-electronic Engineering, National Dong Hwa University, Hualien 97401, Taiwan; u9925044@ems.ndhu.edu.tw (C.-K.S.); u9925001@ems.ndhu.edu.tw (Y.-T.C.); 410025023@ems.ndhu.edu.tw (C.-W.C.); 410325022@gms.ndhu.edu.tw (Y.-R.L.); 410325003@gms.ndhu.edu.tw (J.-S.J.); 410225039@gms.ndhu.edu.tw (Y.-C.C.)

**Keywords:** graphene oxide, oxidation, photodetector

## Abstract

Oxygen-containing functional groups in graphene oxide (GO), a derivative of graphene, can widen the bandgap of graphene. In this study, we varied the amount of hydrogen peroxide used to prepare GO samples with different degrees of oxidation. Transmittance measurement, Raman spectroscopy, and X-ray photoelectron spectroscopy were used to completely characterize the change in oxidation degree. The effects of oxidation degree on p-type and n-type Si heterojunction photodetectors were compared. Notably, GO with a lower oxidation degree led to a larger photoresponse of p-type Si, whereas that with a higher oxidation degree achieved a larger photoresponse of n-type Si.

## 1. Introduction

Heterojunction photodetectors combine two types of materials for light detection, and Si is the optimal choice for one of the two materials due to mature and cheap Si complementary metal–oxide–semiconductor (CMOS) technology with Si acting as the substrate [1,2,3]. Numerous materials have been investigated to be combined with the Si substrate in heterojunction photodetectors for the optimization of photodetector applications, and studies increasingly concentrate on graphene-based materials [4]. The common graphene-based materials in Si heterojunction photodetectors are composed of graphene/Si [5,6,7] or reduced-graphene-oxide (rGO)/Si [8,9,10] structures. In these studies, graphene or rGO is typically only used as the electrode in Schottky diodes. The absorption of graphene itself can also be utilized for certain structures [11] or certain wavelengths [12], but the gapless properties of graphene (and ideal rGO) still restrict its applications in optoelectronic devices [13]. Graphene, with attached oxygen-containing functional groups, is known as graphene oxide (GO) [14]. These oxygen-containing functional groups in GO open the bandgap of graphene. Even partially-reduced GO with suitable manipulation of O/C ratios can still exhibit a large bandgap for utilization in photodetection [15]. The opened bandgap yields more flexibility to graphene-based Si heterojunction photodetectors. For example, an opened bandgap in GO can contribute to light absorption, and enrich the possibility of band engineering. Recently, GO has been integrated on p-type Si substrates (p-Si) to fabricate heterojunction photodetectors [16,17], and GO has also been integrated with n-Si [18,19]. However, to our knowledge, the effects of GO oxidation degrees on the performance of GO/Si heterojunction photodetectors have not been investigated either on p-Si or n-Si. It was reported that the bandgap of GO can vary from 2.3 to 2.7 eV with increasing GO oxidation degrees [20]. Since GO has the advantage of an opened bandgap over graphene, the influence of oxidation degrees is a meaningful subject for GO/Si heterojunction photodetectors. Therefore, the influence of oxidation degrees of GO on Si heterojunction photodetectors is studied in this manuscript. Furthermore, the comparison between influence of oxidation degrees on GO/p-Si and the influence of oxidation degrees on GO/n-Si reveal different trends. We propose that high-oxidation-degree GO is suitable for GO/n-Si heterojunction photodetectors and low-oxidation-degree GO is suitable for GO/p-Si heterojunction photodetectors. The mechanisms are interpreted on the basis of band diagrams.

## 2. Experiments

We prepared GO powder using the modified Hummers method [21]. The main approach to varying the oxidation degree was changing the amount of hydrogen peroxide (H_2_O_2_) used. The details of the experimental procedures are as follows.

Graphite powder, potassium permanganate (KMnO_4_), and sulfuric acid (H_2_SO_4_, 98%) were mixed at a ratio of 1:3:24 below 35 °C. The resulting solution was divided between two beakers. H_2_O_2_ (35%) was then added to both beakers until its volume fraction reached 1% and 4%. The corresponding solutions were used to prepare samples GO1 and GO4, respectively. The resultant solutions were both diluted with deionized (DI) water and continually stirred. Each solution was filtered, and the residual slurry was washed with HCl (3%) and DI water. The slurry was then dried and ground into powder. The powder was added to the DI water, and tiny flakes of GO could be exfoliated and collected through ultrasonication and centrifugation. GO aqueous suspension was then obtained from the supernatant.

**Figure 1:**
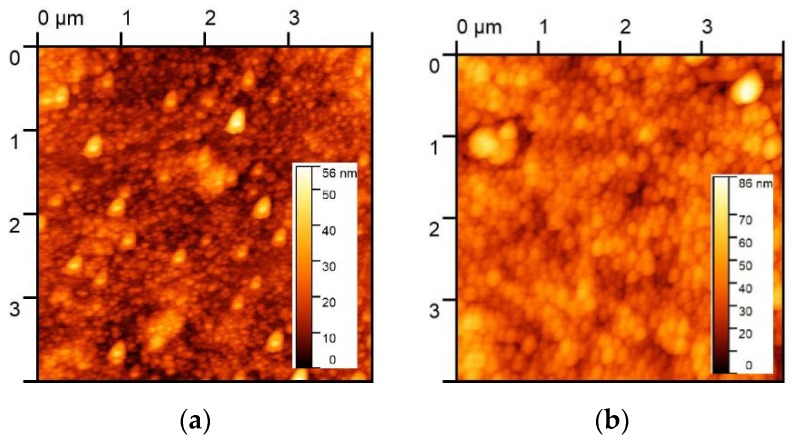
Surface morphology of (**a**) GO1 and (**b**) GO4. GO flakes densely cover the entire surface. Therefore, the difference in electrical characteristics discussed in the text was attributed to the oxidation degree and not to the coverage condition.

To uniformly deposit the hydrophilic GO flakes on Si substrates, the Si substrates were immersed in SC1 solution (1:2:8 NH_4_OH/H_2_O_2_/H_2_O ratio) for 30 min at a temperature of 70 °C [22]. Because of the abundant hydroxyl radicals (OH^−^) in the SC1 solution, the Si substrates also became hydrophilic. Drops of the GO suspension were then applied to the Si surface to form a GO film. Uniform deposition of the GO flakes was possible because of the hydrophilicity of the GO due to its oxygen-containing groups. In our previous study [22], samples treated with SC1 for 0, 15, and 30 min were compared. The longest treatment (30 min) resulted in the lowest tunneling current because of the dense coverage. Because we wanted to form a uniform GO layer, we performed a 30-min SC1 treatment in this study. The morphology of GO1 and GO4 was obtained using atomic force microscopy, as shown in Figure 1a,b. Since both substrates were subjected to the same hydrophilic treatment for 30 min, GO flakes with dense coverage coated the entire surface. The difference in electrical characteristics (which is discussed later) may be attributed to the oxidation degree, but not to the coverage condition.

After GO deposition, a circular Al gate with an area of 5 × 10^−4^ cm^2^ was evaporated on the top of the Si substrate, and a large area of Al was evaporated on the back side of the Si to form an ohmic contact. Both p-type and n-type Si substrates were used to fabricate GO-on-p-Si and GO-on-n-Si heterojunction photodetectors. Current-voltage (IV) measurement was performed using a semiconductor analyzer (Agilent B1500A, Santa Rosa, CA, USA).

## 3. Characterization of Oxidation Degree

**Figure 2:**
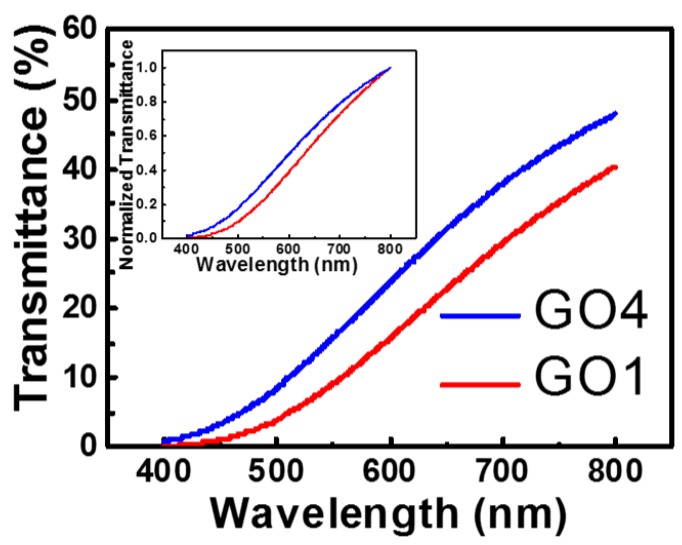
Transmittance of GO aqueous solutions with different oxidation degrees. The inset presents the normalized curves. The onset for GO4 is shifted to shorter wavelengths than those for GO1.

A study found that a higher degree of oxidation resulted in a larger bandgap of GO [20]. To verify whether our procedure changed the oxidation degree, we first performed a spectral absorption measurement (Figure 2). For short-wavelength light, the energy of photons was larger than the GO bandgap, and GO absorbed the light, resulting in a low transmittance. Conversely, GO did not absorb long-wavelength light, which resulted in a high transmittance. Therefore, the onset of the transmittance increase was directly related to the GO bandgap. As shown in Figure 2, the onset for GO4 was shifted to wavelengths shorter than the wavelengths for GO1. This result implies that the bandgap of GO4 was larger than that of GO1. More hydrogen peroxide used in the modified Hummers method resulted in a higher degree of oxidation, which contributed to the larger bandgap.

**Figure 3:**
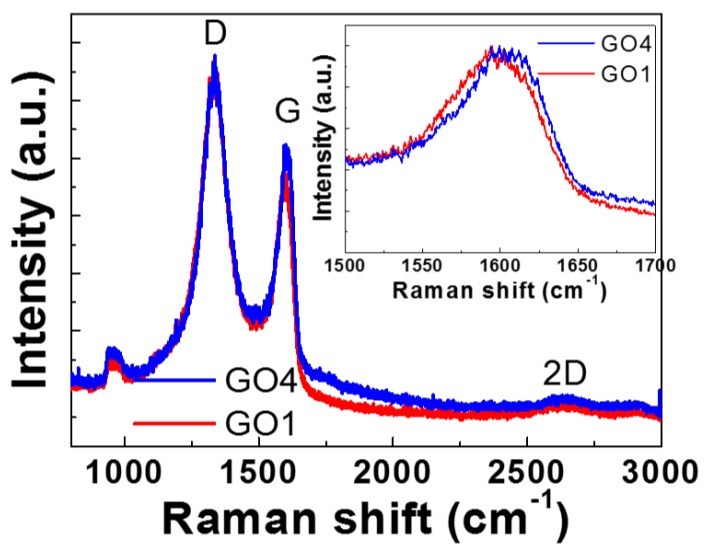
Raman spectra of the GO1 and GO4 samples. A magnified view of the G band is provided in the inset. The excitation wavelength in Raman measurements was located at 632.8 nm. The G peak of GO4 shifted to a higher wavenumber than that of GO1.

Raman spectroscopy is a powerful tool for analyzing the chemical composition and nanostructure of GO [23,24]. The Raman spectroscopy results of GO1 and GO4 are shown in Figure 3. Three bands are typically used to identify graphene-based materials. The vibration caused by defects in the material and disorder at the edge is called the D band, which is located at approximately 1350 cm^−1^ [23,25]. Another band at approximately 2650 cm^−1^, known as the 2D band, is closely related to the number of layers of graphene flakes [23]. Undoped graphene exhibits a peak at approximately 1580 cm^−1^ [26], termed the G band. The position of this band is sensitive to the doping level. Thus, a G-band shift due to the introduction of oxygen, a form of doping, can be an index of the oxidation degree [27].

A magnified view of the G-band peaks of the two samples is shown in the inset of Figure 3. The peak shifted to a higher wavenumber from GO1 to GO4. As observed in a related study [23], the higher oxygen-to-carbon ratio led to a larger Raman shift of the G band. Therefore, it was suspected that the GO4 sample, which had a larger G-band Raman shift, corresponded to a higher oxygen-to-carbon ratio.

**Figure 4:**
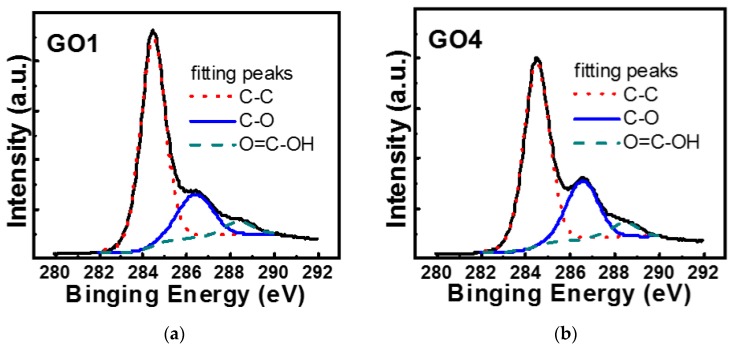
C1s XPS results for (**a**) GO1 and (**b**) GO4. The XPS intensity ratio of C–O (at 286.5 eV) to C–C (at 284.5 eV) bonds of GO4 was larger than that of GO1.

X-ray photoelectron spectroscopy (XPS) provided direct evidence of the difference in H_2_O_2_ treatment resulting in differences in oxygen content. Figure 4a,b show the C1s XPS results for GO1 and GO4, respectively. The well-known binding energy of sp^2^ and sp^3^ C (C–C peak in Figure 4) was at 284.5 eV [22,28]. In addition, the reported binding energies of C–O and O=C–OH bonds matched those from our peak fitting (~286.5 and 288.4 eV, respectively; Figure 4) [28,29]. The XPS intensity ratios of C–O (at 286.5 eV) to C–C (at 284.5 eV) bonds for GO1 and GO4 were 0.28 and 0.40, respectively. These indicated that the oxygen content in GO4 was higher than that of GO1.

## 4. Electrical Characteristics

**Figure 5:**
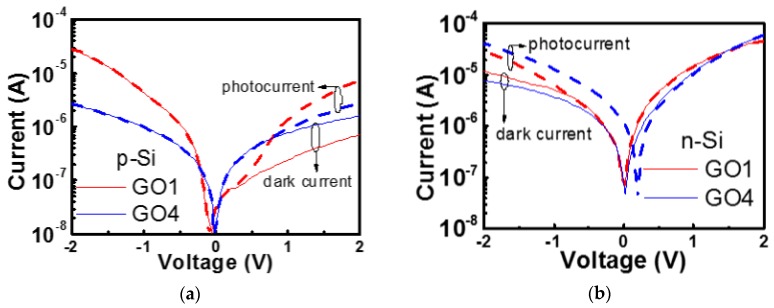
Dark currents and photocurrents of GO1 and GO4 heterojunction photodetectors on (**a**) p-Si and (**b**) n-Si. The photocurrents were measured under white light from white light emitting diodes with a power density of 0.5 mW/cm^2^.

Si substrates with GO1 and GO4 deposited on the surface were used to fabricate GO1 and GO4 photodetectors, respectively. GO flakes and the native oxide comprised the oxide layers. The photocurrents and dark currents of p-Si and n-Si heterojunction photodetectors were measured, as shown in Figure 5a,b.

**Figure 6:**
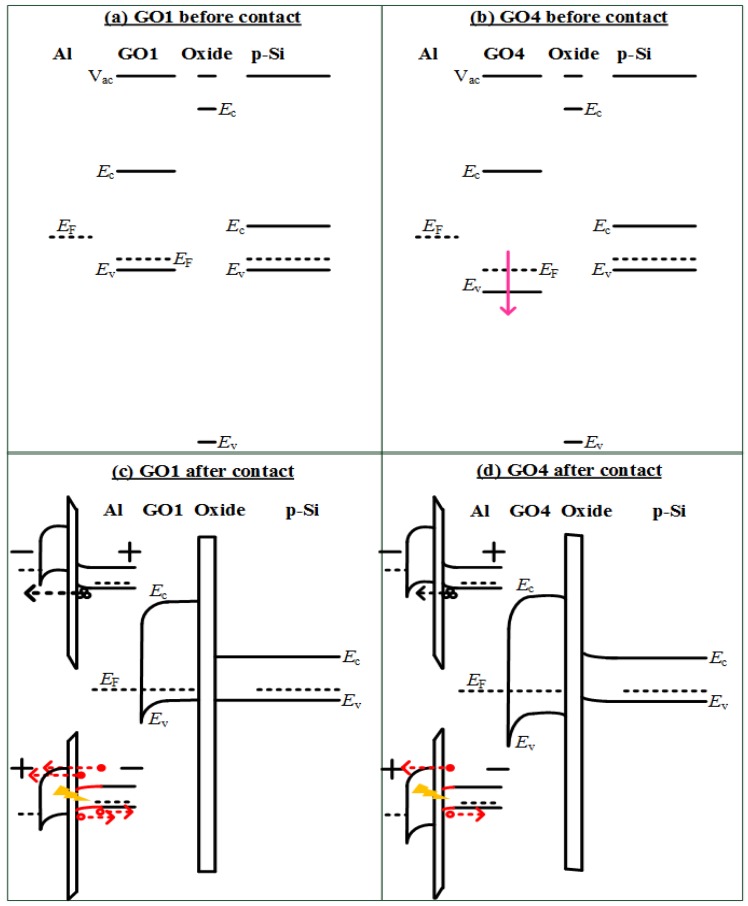
Band diagrams for (**a**) GO1 and (**b**) GO4 detectors on p-Si before and (**c**) GO1 and (**d**) GO4 detectors after contact. The larger work function of GO4 was detrimental to the formation of a depletion region in the p-Si.

**Figure 7:**
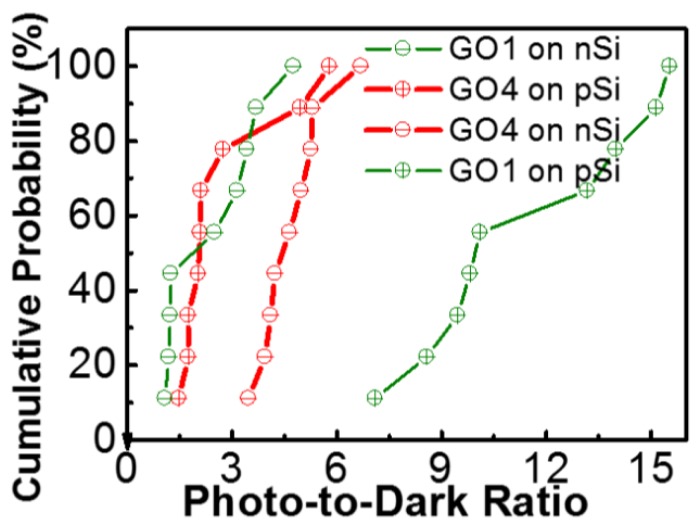
Cumulative probability plot of photo-to-dark ratios. On p-Si, GO1 results in larger ratios than those of GO4, whereas GO1 leads to smaller ratios on n-Si.

A higher oxidation degree corresponds to a larger bandgap [20]. Schematic band diagrams of our p-Si detectors with GO1 or GO4 (Figure 6) were constructed according to the data in [20]. A higher oxidation degree of GO4 resulted in a larger work function and bandgap compared with those of GO1. In [20], the conduction band edge (*E*_C_) did not change but the valence band edge (*E*_V_) was lowered as the oxidation degree increased. Therefore, a downward shift of *E*_V_ was present regarding GO4 relative to that in GO1 of our photodetectors (Figure 6d as compared with Figure 6c), which resulted in a large barrier for holes from Si to the Al gate at the accumulation (negative) bias. This is illustrated in the upper insets of Figure 6c,d. The hole flow from Si to Al might have been blocked by GO4 in the GO4 device (the upper inset of Figure 6d), whereas more hole flow from Si could reach the Al gate successfully in the GO1 device (the upper inset of Figure 6c). Therefore, the accumulation current of GO4-on-p-Si was smaller (Figure 5a). There might have been some electron flow from the Al gate to Si that overcame the *E*_C_ barrier at the accumulation bias. However, this component was small for the GO1 and GO4 cases because the *E*_C_ barrier of GO was large and thick. Therefore, the GO4-on-p-Si photodetector exhibited a smaller accumulation current than that of GO1-on-p-Si mainly due to the larger barrier for the holes from the Si to the Al gate. Photodetection should be performed at the inversion bias (positive bias in the p-substrate case) because the high electric-field in the depletion region at the inversion bias could help the carrier separation of photo-generated electron-hole pairs. The band alignment suggested that the larger work function of GO4 was detrimental to the formation of a depletion region in the p-Si. The Fermi level (*E*_F_) of GO4 was lower than the *E*_F_ of p-Si, resulting in initial accumulation (the Si band at the surface was oriented upward at 0 V) (Figure 6d). At the positive bias, the initial accumulation of the GO4-on-p-Si resulted in a thinner depletion region (red solid line on the lower inset of Figure 6d) compared with the thicker depletion region of GO1-on-p-Si (red solid line of the lower inset of Figure 6c). Thus, the photocurrent of the GO4-on-p-Si was smaller than that of the GO1-on-p-Si. The photo-to-dark ratio of the GO4-on-p-Si was 1.75, which was smaller than that of the GO1-on-p-Si at 2 V (10.1). At 2 V, the absolute photoresponse (difference between the photocurrent and dark current) of the GO4-on-p-Si (1.2 × 10^−6^ A) was therefore smaller than that of GO1-on-p-Si (6.4 × 10^−6^ A). To validate these findings, more samples were prepared and measured. The cumulative probability plot of photo-to-dark ratio is shown in Figure 7. On p-Si, all the GO4 photodetectors exhibited smaller photo-to-dark ratios than those of the GO1 photodetectors.

Figure 5b shows the IV behaviors of n-Si photodetectors. On n-Si, the accumulation bias (positive bias in the n-substrate case) drove electrons from the Si to the Al gate and the holes from the Al gate to the Si. In the n-Si photodetector, the *E*_C_ barrier for electrons from the n-Si to Al gate formed by GO became smaller than the *E*_C_ barrier from the Al gate to the p-Si in p-Si photodetectors. Therefore, the electron flow could not be ignored now. The electron flow will considerably contribute the total current in the n-Si photodetectors.

**Figure 8:**
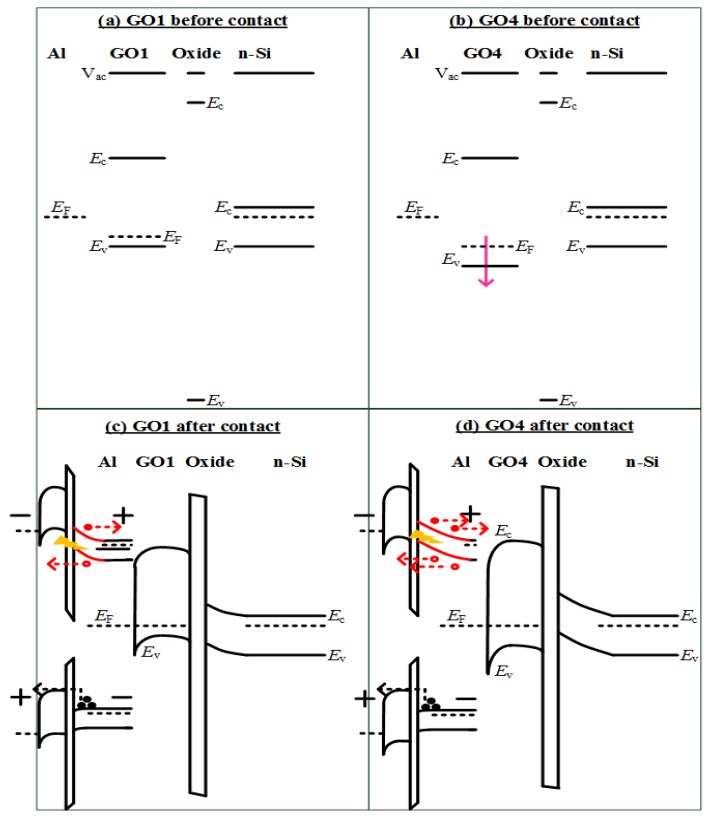
Band diagrams for (**a**) GO1 and (**b**) GO4 detectors on n-Si before and (**c**) GO1 and (**d**) GO4 detectors after contact. At the inversion bias (upper inset of (**c**,**d**)), the larger work function of GO4 contributed to a wider depletion region in the n-Si. At the accumulation bias (lower inset of (**c**,**d**)), the component of “electrons from Si to the Al gate” would predominate over the component of “holes from the Al gate to Si” due to the smaller SiO_2_ oxide barrier.

The accumulation current of the GO4-on-n-Si photodetector, therefore, displayed no obvious difference with that of the GO1-on-n-Si photodetector, because the barrier for electrons from n-Si to the Al gate in the GO4-on-n-Si photodetector (lower inset of Figure 8d) was similar to that in the GO1-on-n-Si photodetector (lower inset of Figure 8c). Oxidation degrees mainly influenced the *E*_V_ and *E*_F_, but not the *E*_C_ [20] as shown in Figure 8a,b. However, the relation between the photoresponse at the inversion bias (negative bias in the n-substrate case) and oxidation degrees in the n-Si case was the inverse of that of the p-Si case. The photo-to-dark ratio of the GO4-on-n-Si was 5.3, which was larger than that of the GO1-on-n-Si at −2 V (2.5). The absolute photoresponse of the GO4-on-n-Si at −2 V (3.4 × 10^−5^ A) was also larger than that of the GO1-on-n-Si (1.8 × 10^−5^ A). Notably, GO4 provided a superior photoresponse than GO1 on n-Si, whereas GO4 led to an inferior photoresponse on p-Si. This substantial photoresponse of the GO4-on-n-Si occurred because the larger work function of GO4 contributed to a thicker depletion region (red solid line of the upper inset of Figure 8d) in n-Si compared with the thinner depletion region of the GO1-on-n-Si (red solid line of the upper inset of Figure 8c). Therefore, the cumulative probability plot demonstrated that the photo-to-dark ratios of the GO4-on-n-Si photodetector were significantly larger than those of GO1-on-n-Si (Figure 7).

## 5. Conclusions

GO-on-p-Si and GO-on-n-Si heterojunction photodetectors were comprehensively studied. Different oxidation degrees of GO were achieved by varying the amount of hydrogen peroxide used in the oxidation procedure of GO. Our results indicated that GO with lower oxidation degrees should be applied to p-Si heterojunction photodetectors to achieve superior photoresponses, whereas GO with higher oxidation degrees was applicable for n-Si heterojunction photodetectors. Oxidation degrees led to opposing influence on GO/p-Si and GO/n-Si photodetectors, and the corresponding influence on the depletion region provided the possibility of photodetector optimization through such band engineering.
